# Using the frame averaging of aS500 EPID for IMRT verification

**DOI:** 10.1120/jacmp.v4i4.2499

**Published:** 2003-09-01

**Authors:** J. Chang, C. C. Ling

**Affiliations:** ^1^ Medical Physics Department Memorial Sloan‐Kettering Cancer Center 1275 York Avenue New York New York 10021

**Keywords:** amorphous silicon, EPID, IMRT, IMRT treatment plan verification

## Abstract

In this study, we evaluated the use of aS500 EPID for the verification of IMRT beam delivery, using the synchronous, frame‐averaging acquisition. In this approach, an EPID continuously integrates frames while irradiated by an IMRT field; the averaged image is then converted to a dose profile using a linear calibration curve, and is compared with the planned profiles using a linear‐regression model, which returns an index σ (root mean squared error) for the goodness of fit. We identified several potential errors in this acquisition mode: missing data between the start of irradiation and imaging, and from the last (incomplete) frame, which we proved are insignificant for IMRT fields; and EPID dead time during irradiation stemming from data transfer, which we successfully corrected for clinical MU (>100). We compared the measured relative profiles and central axis dose of 25 prostate fields with the planned ones. Applying our correction methods, very good agreement was obtained between the measured and planned profiles with a mean a of 1.9% and a standard deviation of 0.5%; for central‐axis dose the agreement was better than 2.0%. We conclude that the aS500 is an effective tool for verification of IM beam delivery in the range of clinical MU (>100) settings. Although the vender is developing an upgrade to fix similar problems, our results demonstrate that the current configuration with simple correction schemes can achieve satisfactory results.

PACS number(s): 87.53.Oq, 87.53.Xd

## INTRODUCTION

The application of electronic portal‐imaging device (EPID) for intensity modulated radiotherapy (IMRT)^1^ ^,^ ^2^ verification has been studied by us^3^ ^–^ ^5^ and many others.^6^ ^–^ ^10^ With the use of a scanning liquid‐filled ionization chamber (SLIC) or charge‐coupled device (CCD) camera based EPID, typically accuracy of 3% in central axis dose can be achieved, better than the 5% requirement recommended by the Task Group 40 report^11^ of the American Association of Physicist in Medicine (AAPM) for independent verification of the dose at the isocenter or at a point. We also showed^3^ that the root mean‐squared difference between the planned and measured profiles for the in‐field region was within 5%. We note that there is as yet no AAPM recommendation for the accuracy of relative profile verification.

Although this is sufficiently accurate for clinical quality assurance (QA), the slow response, detector memory effects, and beam hold‐off problem make it impractical for routine IMRT verification using the SLIC EPID.^5^ The camera‐based EPIDs suffer from low light collection efficient;^12^ optical glaring errors^13^ ^,^ ^14^ also complicates the use of these devices for dosimetry verification. Recent development in amorphous‐silicon EPID has made it a device of choice for radiotherapy. Its imaging speed can be as high as ten frames per second. When operating at the clinical dose rate, the detector has a linear response as a charge accumulation device; therefore, dose integration can be provided by the frame‐averaging. By setting a large number of frame averages, all acquired frames are summed, and an averaged image is sent to the console computer upon completion of radiation delivery. Without network overhead, the maximum image speed is available for verification purposes. Munro and Bouius also demonstrate that this EPID does not suffer from the glare phenomenon associated with the camera‐based EPID.^15^

In the future, there will be two versions of software available to control the image acquisition. The “current” version, e.g., the PortalVision system release 6.0.56 used in this study, is used by existing aS500 EPID; however, when it is used for IMRT verification, a number of frames are lost due to detector dead time, leading to artifacts and an underestimate of measured dose. The manufacturer is developing an upgrade^16^ to fix these problems, though it is still in the beta testing stage and is not available for most users, including us. In this study, we only used the current version and developed schemes to correct the problems encountered during IMRT verification. As we will show in this paper, the methods that we have developed are immediately applicable to the many aS500 EPIDs that are in clinical implementation. Our methods should also work for the upgraded EPIDs, and thus provide an alternative approach different from that of the vendor.

In this study, we evaluate the use of aS500 EPID for verifying the relative profile and central‐axis (CAX) dose of IM beams without phantom, and assess the effects of these errors on the verification results. We investigate the dose integration approaches for converting the averaged reading to dose and identify correction factors that are required. We test the proposed integration approaches and the correction factors by verifying the relative profiles and absolute doses of 25 IMRT fields.

## METHODS

### A. aS500 EPID

Varian's aS500 EPID consists of a buildup layer, an amorphous‐silicon detector panel, readout electronics, and a PortalVision workstation. The buildup layer consists of a 1‐mm copper plate for electron production, and a scintillating phosphor screen (0.34‐mm ${\text{Gd}}_{2}O_{2}S:\text{Tb}$, 0.52‐mm for the entire screen) for conversion to visible light. The detector panel is a matrix (512×384) of individual light‐sensitive photodiodes for integrating the light, and a thin‐film transistor, the switch to the readout electronics. The sensitive area of the detector panel is 40×30 cm^2^, corresponding to a pixel size of 0.78 mm. Each row of the detector matrix is sequentially scanned by the readout electronics.

There are two modes to read out the detector signals. In the synchronous mode, the detectors are read out in sequence during the time intervals between the radiation pulses; in the asynchronous mode, the readout is controlled by an internal clock. In either case, the entire matrix can be scanned in ~0.111 s. The asynchronous acquisition is intended for a continuous radiation source that has no beam pulse to clock the scanning; when used with a pulsed radiation source, streak artifact is observed because the radiation pulse adversely affects the readout electronics. The synchronous acquisition minimizes this artifact by scanning each row at fixed time after a pulse, and by applying the flood image correction. Given the fact that synchronous acquisition can achieve the same high imaging speed without significant streak artifacts, and is the default acquisition mode in aS500, we use the synchronous mode only in this study.

We define a complete read‐out of the matrix as a frame, which can be immediately exported or stored in a buffer for integration with the next frame. In routine operation such as port filming, a number of (e.g., five) frames are averaged to improve the image quality.

### B. Frame averaging

**Figure 1:**
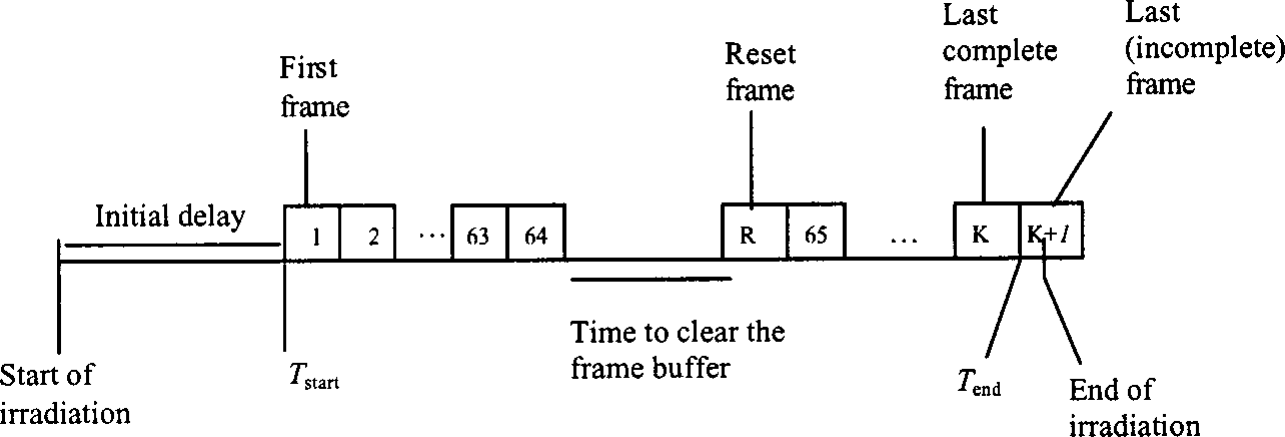
Illustration of the timing and detector‐dead time for image acquisition using the frame‐averaging mode. Frames are sequentially acquired every 0.111 s, and are integrated in the frame buffer; the integrated reading is divided by the number of complete frames, *K*, to obtain the averaged reading. To avoid saturation, data in the frame buffer are exported every 64 frames, followed by a reset frame, both of which are not averaged. The total detector‐dead time includes the initial delay, time to clear the frame buffer and following reset frames, and the last incomplete frame. Initial delay, the time between switching the beam on and starting image acquisition $\left(T_{\text{start}}\right)$, includes the start delay and the reset frames before the first frame. $T_{\text{end}}$ is the finishing time for the last complete frame. The last incomplete frame is not included in the frame averaging.

Frame averaging (Fig. 1) is an acquisition configuration of aS500 by which “complete” frames are continuously acquired from the start of the irradiation till the end, integrated in a buffer, and then averaged. A frame is “complete” if every pixel is exposed to the radiation for the same amount of time between two scans. Note that the initial delay in Fig. 1 includes two adjustable EPID settings: the “Start Delay” and the “Number of Reset Frames.” Although both can be set to zero, at least one reset frame is needed to clear the buffer before image acquisition; otherwise, the first frame is not complete. For better image quality in routine portal imaging, one sets either a “Start Delay” of a few tenths of 1 s followed by one reset frame, or several (e.g., three to five) reset frames with a zero “Start Delay.” The imager also discards the last incomplete frame, of which the irradiation ends in the middle.

Since the frame buffer can only integrate up to 64 frames before saturation, the integrated data need to be transferred periodically to another storage area. As shown in Fig. 1, it takes ~0.164 s for the transfer and another 0.111 s for reset. Thus, there is a dead time of 0.275 s every 7.10 s (the time for acquiring 64 frames). We note that with the upgrade version the EPID can scan the charges accumulated in the last incomplete frame and capture the information during the clearing of the frame buffer, which is not feasible with the current version.

### C. Converting the frame‐averaged reading to dose

The number of complete frames (*K*), from which the average EPID reading was obtained, is returned with the averaged image, and can be used to convert the averaged EPID reading to the accumulated dose. Theoretically, the total dose, D, is the integration of the dose rate, *D*(*t*):

(1) $$D=\sum_{k=1}^{K}\int_{T_{k}}^{T_{k+1}}\dot{D}(t)dt+D_{B}+D_{SE},$$

where $T_{k}$ is the starting imaging time for frame *k*, $D_{B}$ represents the “missed” doses delivered during the buffer clearing, and $D_{SE}$ is the sum of doses delivered before $T_{\text{start}}$ and after $T_{\text{end}}$. Since the dose between $T_{k}$ and $T_{k+1}$ is integrated by frame k, we can rewrite Eq. (1) as

(2) $$D=K\times \left(\frac{1}{K}\sum_{k=1}^{K}D_{k}\right)+D_{B}+D_{SE}=K\times D_{avg}+D_{B}+D_{SE}=K\times \left(\frac{R_{avg}}{a}\right)+D_{B}+D_{SE},$$

where $D_{k}=\int_{T_{k}}^{T_{k+1}}\dot{D}(t)dt$ is the dose for frame *k*, and $D_{avg}$ is the averaged dose converted from $R_{avg}$ using R=aD, the linear dose calibration curve characterized by the coefficient *a*.

### D. Correction for the missing dose during buffer clearing

As we shall prove later, $D_{SE}$ is insignificant for IMRT fields; $D_{B}$, on the other hand, accounts for ~4% of the delivered dose and needs to be corrected. For a given *K*, the recorded imaging time is 0.275×⌊(K−1)/64⌋ s, longer than the actual imaging time, K×0.111, where “⌊⌋” is the floor function. To correct for this loss of the dose, a correction factor $CF_{fb}$ is added to Eq. (2),

(3) $$D=CF_{fb}\times K\times \frac{R_{avg}}{a}+D_{SE},$$

where

(4) $$CF_{fb}=1+[\left\lfloor (K-1)/64\right\rfloor \times 0.275]/(K\times 0.111),$$

or if K/64≫1, can be approximated as

(5) $$CF_{fb}\approx 1+0.275/(64\times 0.111)=1.039.$$

Although $D_{B}$ can be easily corrected for static fields using Eq. 4(a), it cannot be totally removed for IMRT fields, because all pixels are not irradiated at the same time, resulting in an uncertainty in the number of buffer clearances a pixel has experienced when it is in the open part of the field. However, as shown later, the MU numbers are high enough for clinical IMRT field so that Eq. 4(b) can be safely used. For the “upgrade” configuration, $CF_{fb}$ is reduced to unity because the dose is collected during the buffer clearing.

### E. Relative profile and absolute dose verification

We have previously developed a QA method^3^ using a SLIC EPID for verifying the relative profile of the in‐field region and absolute dose of IMRT fields. In this method, additional buildup is placed on the EPID for higher (e.g., 15 MV) energy, in order to achieve the electron equilibrium for dose measurement. The measured dose profile *M*[*i,j*] is compared with the planned dose profile *I*[*i,j*]:

(6) M[i,j]=c×I[i,j]+s+E[i,j],

where *s* is a correction factor for phantom (or patient) scatter, *c* is a normalization constant, and *E*[*i,j*] is the error matrix. Using the linear regression^17^ ^,^ ^18^ approach, we optimize *s* and *c* to minimize the mean square error $\sigma^{2}=\sum_{i,j}E{\left[i,j\right]}^{2}/(N-1)$ where *N* is the total number of pixels. The minimized σ is a measure of the goodness of fit between the planned and measured dose profiles. For absolute dose verification, the dose at the prescription depth is calculated using the pencil beam convolution^19^ and TMR ratio:

(7) $$D_{d}[i,j]=M_{SAD}[i,j]⊗\kappa_{d}[i,j]\times [TMR(d,r_{p,SAD})/TMR(d,10\times 10)],$$

where $D_{d}[i,j]$ is the dose map at depth *d*, $M_{SAD}[i,j]$ the measured profile at SAD, $\kappa_{d}[i,j]$ the convolution kernel for depth *d*, and $r_{p,SAD}$ the equivalent square field size at SAD. $M_{SAD}[i,j]$ is derived from *M*(*i,j*] using back‐projection, corrected for inverse square and EPID phantom scatter factor $\left(S_{pe}\right)$ ^(3)^: $M_{SAD}[i,j]=BP(M_{SDD}[i,j])\times {\left(SDD/SAD\right)}^{2}/S_{pe}(r_{p,SDD})$, where *BP* is the back projection operator, and $r_{p,SDD}$ the equivalent square field size at the source to detector distance, *SDD*.

## EXPERIMENTAL MEASUREMENTS

All experiments were conducted at a SDD of 150 cm and using a 15 MV beam at a dose rate of 300 monitor units (MUs) per minute. We adopted the “regular quality scanning mode” used for port filming −0.111 s per frames, 0 ms for “Start Delay,” and five reset frames before acquiring the first frame, corresponding to an initial delay of 0.555 s; and set the number of frames averages to 5000. Note that it will take ~550 s to acquire 5000 frames, longer than the time required for irradiation; however, the frame average stops when the beam is turned off; thus, <5000 frames were averaged. The first experiment was to determine the additional buildup layer needed to achieve the electron equilibrium for the 15 MV beam. Subsequently, this additional buildup of a 1.5 cm thick polystyrene plate was always added to the EPID.

We then determined the calibration curves and the phantom scatter factor of the EPID, $S_{pe}$, for different field sizes. Definition and measurement of $S_{pe}$ are described in details in Ref. 4. For each field size, we manually acquired images (0.111 s per frame, five frame averages) every 10 s after the dose rate of the LINAC was stabilized, for six images. $S_{pe}$ of a given field size was the ratio of the averaged reading of the central 1×1‐cm^2^, to that of the 10×10‐cm^2^ field, divided by the collimator scatter factor. For comparison, the phantom scatter factors of a full water phantom, $S_{p}$, and of a slab water phantom (with full build up and no backup), $S_{ps}$, were also measured for different field sizes, using a pancake ionization chamber. We used Khan's formalism^20^ for the definition of phantom scatter factor.

For the dose calibration curve, the EPID was irradiated with various radiation intensities achieved using different SDD (every 10 cm from 100 cm to 160 cm) or different amount of lead attenuation (every 6.3 mm from no lead to 70‐mm lead); the averaged CAX readings of the acquired images were obtained using the procedure described above. The dose rate for each radiation intensity was measured using a Spokas ionization chamber in a mini‐phantom (3‐mm copper), multiplied by $S_{pe}$ of the corresponding field size. The calibration curve was determined by plotting the averaged CAX reading against the measured dose rate multiplied by 0.111 s.

To illustrate the effect of buffer clearing, we irradiated the EPID with two IM fields: a 10‐mm sliding window IMRT field and a prostate IMRT field, with different (from 5 to 1000) MU settings. The number of frames (*K*) and imaging time $\left(T_{\text{img}}\right)$ were recorded for each irradiation; and the dose profiles converted from the EPID readings were compared with the planned profile. For the prostate IM field, an initial delay of 0.111 s (one reset frame before acquiring the first frame) was also tested for comparison.

We also used 25 prostate IMRT fields from five treatment plans (five fields for each plan) to evaluate the EPID's capability for verifying the IM profiles. We acquired the frame‐averaged images of these IMRT fields, and converted them to dose profiles using the calibration curve. Three (100, 130, and 350) MU settings were tested for each field. The 100 and 130 MU settings correspond to the lower and average MU settings of the 25 fields. Because significant beam hold‐off (the withholding of LINAC beam pulses when MLC leaves are not in the correct positions) was observed for both 100 and 130 MU settings, the 350 MU setting was added to study the effect of beam hold off.

The in‐air fluence profiles for the same IM fields were calculated using the treatment planning system, and were convolved with a Gaussian kernel (2.3‐mm full width at half maximum) to obtain the planned dose profiles at the dose max $\left(d_{\text{max}}\right)$ of the EPID. The kernel was experimentally determined by iteratively adjusting its size to optimize the fit between the measured and planned dose profiles of a pre‐selected IMRT field. The measured and planned profiles were then compared using the method previously described. The measured CAX dose, the sum from the five IM fields weighted by planned beam‐on time and TMR at the treatment depth, was also compared with the planned dose.

## RESULTS

**Figure 2:**
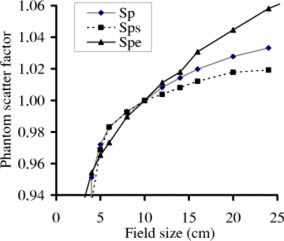
(Color) The phantom scatter factor ($S_{pe}$) of an aS500 EPID is shown for the 15 MV beam of a Varian 2100EX LINAC. Phantom scatter factors of a full water phantom ($S_{p}$) and a slab water phantom ($S_{ps}$) are also shown for comparison.

Figure 2 shows the phantom scatter factor of the EPID, $S_{pe}$, which is more pronounced than those of a full water phantom ($S_{p}$) and a slab phantom ($S_{ps}$).

**Figure 3:**
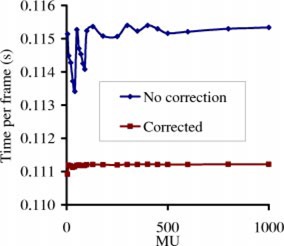
(Color) Time per frame vs given MU for an IMRT field. Time per frame is calculated as the imaging time $\left(T_{img}\right)$ divided by the number of frames (*K*).

We show in Fig. 3 the imaging time per frame $\left(T_{img}/K\right)$ as a function of MU for an IMRT field, with/without using Eq. (4a) to correct for the missing frames. Without correction, $T_{img}/K$ varies significantly (up 2%), especially at small MU settings, due to data transfer from the frame buffer every sixty‐four frames. The uncorrected $T_{img}/K$ approaches an asymptotic value of 0.115 s/frame, higher than the corrected value of 0.111 s/frame by ~3.6%, similar to the predicted 3.9% in Eq. (4b). Notice that, during the image acquisition, the dose rate of the LINAC is 274 MU/min somewhat (~8%) lower than the nominal dose rate of 300 MU/min because the EPID regulates the LINAC dose rate to optimize image quality.

**Figure 4:**
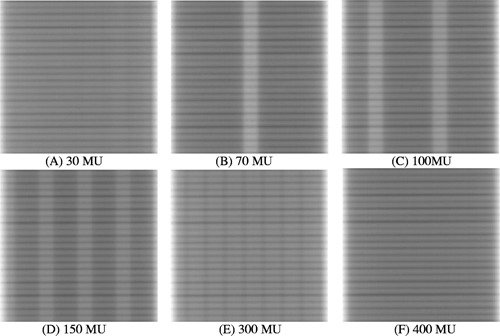
Images of a 10‐mm sliding window IMRT field for six different MUs, acquired using the frame averaging of an aS500 EPID. The leaves move from the right side of image to the left. A darker pixel corresponds to a higher dose.

Figure 4 shows selected images of the 10‐mm sliding window IMRT field for six different MUs. This IMRT field is intended to produce a spatially uniform fluence. Since the total imaging time (6.6 s) for the 30‐MU case is less than 7.1 s, no detector dead time is observed in panel (A). The detector dead time produces white‐and‐dark vertical band artifacts for larger MU settings, as shown from panels (B)‐(E), with higher MU setting for more bands; no significant band patterns are observed for MU>400, as shown in panel (F).

**Figure 5:**
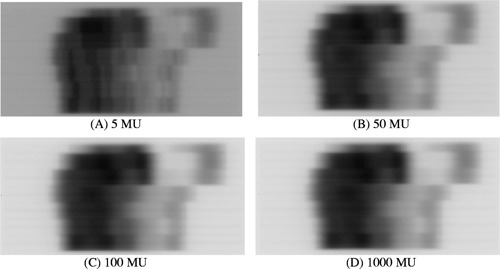
Images of a prostate IMRT field for four different MUs, acquired using the frame‐averaging mode of an aS500 EPID. A darker pixel corresponds to a higher dose.

Figure 5 shows the images of a prostate IMRT field for four different MU settings −5, 50, 100, and 1000. The band pattern is clearly observed for the 5‐MU image, but only barely for the 50‐MU case; when the MU is higher than 100, no significant artifacts are observed.

**Figure 6:**
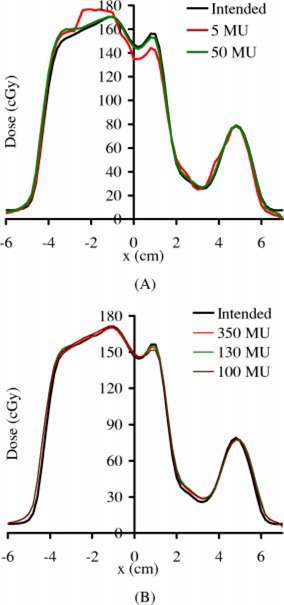
(Color) Transverse beam profiles for (A) 5 and 25 MU, and (B) 100, 130, and 350 MU, of the prostate IMRT field in Each measured dose profile is re‐normalized to the level of the planned dose profile, using the regression coefficients [Eq. (5)].

The effect of higher MU is further demonstrated in Figs. 6(a) and 6(b), where the measured transverse dose profiles for (a) 5 and 25 MU, and (b) 100, 130, and 350 MU, of the prostate field in Fig. 5 are plotted. Although significant deviation from the planned dose profile is observed for the 5‐MU case in Fig. 6(a), the 50‐MU curve is reasonably close to the planned; there are essentially no differences between the planned and the measured profiles for all MU settings in Fig. 6(b).

**Figure 7:**
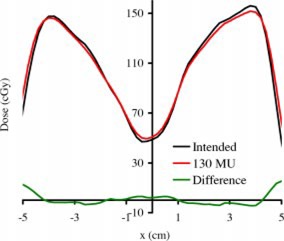
(Color) Transverse beam profiles for another field for the same plan as used in not corrected using the regression coefficients (Eq. 5); the difference (measure–planned) profile is also plotted. The average difference is 1.78% (normalized to the average measured dose of the in‐field region). Twenty‐five percent of the pixels are more than 3% difference in dose; however, they are all within 3 mm (isodose shift).

Figure 7 shows the transverse beam profiles of the in‐field region for another field of the same plan used in Fig. 5, not corrected using the regression coefficient [Eq. (5)]. The doses of all the pixels are with 3% difference or with 3 mm (isodose shift line).

**Figure 8:**
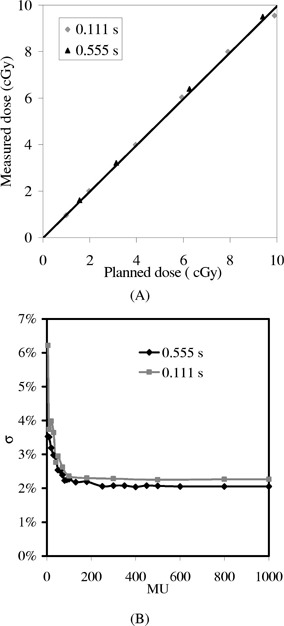
(A) Measured vs planned CAX dose for the IMRT field shown in Fig. 5 corrected using Eq. (4a), for different initial delay settings −0.111 s and 0.555 s. Only results for the low dose settings are shown although dose settings ranging from a few cGy to a few hundred cGy were tested. The best linear fits are y=0.997x−0.016 and y=0.997x−0.042 for “0.111 s” and “0.555 s,” respectively. (B) The results of relative profile verification, σ vs MU, of the same IM field.

In Fig. 8(a) we show the measured dose [corrected using Eq. (4a)] versus the planned dose for the prostate IMRT field in Fig. 5 with two different initial delays (0.111 s and 0.555 s). No significant differences are observed between these two curves acquired with different initial delays. Figure 8(b) plots the results for profile verification, σ versus MU of the IMRT field for the same initial delay settings; the σ values of both curves drop to ~2.1% for MU>200. The good agreement between these two initial delays indicates that $D_{SE}$ is insignificant for the tested IMRT fields.

**Figure 9:**
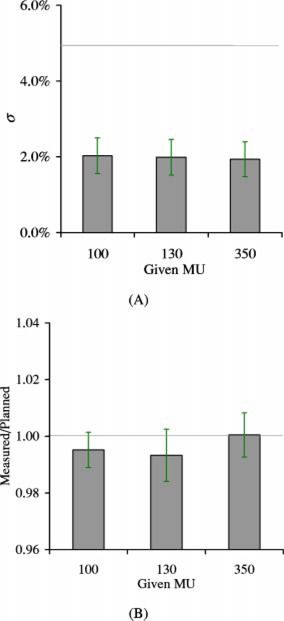
(Color) Comparison of IMRT verification results for 100, 130, and 350 MUs, using the aS500 EPID for 25 prostate fields. (A) Relative profile verification: mean σ vs MU, with the horizontal line indicating the accepTable 5% QA tolerance. (B) Mean measured/planned vs MU, i.e., ratio of dose from EPID measurements to that calculated from the treatment planning, corrected using Eq. (4b), with the horizontal line being the ideal ratio (1.0). The error bar is one standard deviation of the averaged σ for measured/planned dose.

Figure 9 compares the results for verifying the relative profiles, and isocenter dose corrected using Eq. (4b), of the twenty‐five IM fields, for 100, 130, and 350 MUs. It is observed that the mean σ+SD in Fig. 9(a) is ~2.5%, much lower than the 5% QA tolerance.

## DISCUSSION

Although the buildup materials are different for the Mark 2 (Plastoferrite) and the aS500 (Copper) EPIDs, both devices are designed for portal imaging with 6 MV photons (buildup=14 mm polystyrene). Thus, the additional ~15 mm polystyrene buildup for the aS500 to achieve electron equilibrium for the 15 MV x‐ray (buildup=31 mm polystyrene), is the same as that required for the Mark 2 SLIC EPID.^3^

Although the lead attenuator may change the beam quality, the effect is of negligible consequence relative to calibration curves of the 15 MV beam as the same slope was obtained for the calibration curve either using lead sheets and inverse square to attenuate the beam. For the 6 MV beam, however, up to 10% difference in slope was observed for the calibration curves obtained using lead sheets and inverse square to attenuate the beam. This is expected because the phosphor screen of aS500 EPID is more responsive to low energy photons, thus more sensitive to the beam hardening effect of the 6 MV beam. The higher phantom scatter factors for the aS500 EPID in Fig. 2 may be due to increased scatter from the buildup, higher detector sensitivity to the low‐energy incident or scattered photons, or the spreading of optical photons created in the phosphor layer. In any case, the field size dependence factor $S_{pe}$ account for all possible contributions, and Eq. (6) can be used to convert the EPID dose to phantom dose.

The identified sources of error (sampling error, beam hold‐off, and detector memory) for the SLIC EPID^3^ ^,^ ^5^ are not as serious for the aS500 EPID. Although we did not thoroughly test the detector memory, we did not observe any significant detector memory effects in the experiments, as we did in Ref. 5. The detector memory effect was observable when the aS500 EPID was irradiated for a large number of MU (a few hundreds); however, the effect was so small (<0.5%) that any attempt to correct it wasn't justified. The beam hold‐off also has a negligible effect on the verification results; as shown in Figs. 9(a) and 9(b), results of relative profile and CAX dose verifications are similar for the beams with significant (on average 18% of the segments) hold off (the 100 and 130 MU settings), and without any hold off (the 350 MU setting).

There is essentially no sampling error, except for the missing 0.275 s of every 64 frames, because the detector actually integrates all doses between two scans. As shown in Figs. 4 and 5, the missing 0.275 s of every 64 frames due to buffer clearing poses a serious problem only for lower MU settings. For higher MU number, the introduced artifacts can be safely ignored for the relative profile verification, as shown in Fig. 8(b); or can be corrected using Eq. (4b) for CAX dose, as shown in Fig. 8(a).

Since the initial delay is usually set to more than one reset frame for other clinical use of the EPID (e.g., portal filming), this parameter will need to be constantly altered if set to a different value for IMRT verification, which may lead to confusion and errors in a busy clinical environment. As shown in Fig. 8, the error from initial delay is insignificant for clinical IMRT fields. Thus, we can use the same configuration setting as other applications and avoid the confusion.

Together with the similar mean σ, mean measured/planned dose and SD for all three MU settings shown in Fig. 9, we can safely infer that, for clinical prostate IMRT fields using 100–150 MU per field, this artifact doesn't significantly distort the measured dose profile, and can be accurately corrected using Eq 4(b). We note that the experience with our prostate IMRT field may not be directly applicable to other IMRT fields; for IMRT fields of other sites, similar tests should be performed.

McCurdy *et al*.^21^ used Monte Carlo simulation to calculate the dose kernel of aS500 EPID, and suggested that the tail of the point spread function needs to incorporate an exponential component arising from optical photon glare. In their study, portal dose images in the phosphor and coupled to the glare model generally allowed prediction to within 5% in low‐dose gradient regions, and to within 5 mm in high‐dose gradient regions of the measured images. The use of experimentally determined Gaussian kernel (2.3‐mm full width at half maximum) in this and previous35 studies to calculate the planned distribution is simpler and equally effective, although the tail of a Gaussian kernel is different from that of an exponential function. It is illustrated in Fig. 7 that the dose differences of all pixels in the in‐field region of the studied IMRT field are within 3% or 3 mm. Analysis of the global data in Fig. 9 also indicates that, 96% of the pixels in the planned profile are within 3% [mean+2 S.D. of Fig. 9(a)] of the measured, and the CAX dose of the planned profile is within 2% of the measured [Fig. 9(b)]. Thus, in the worse case scenario, most of the pixel of the planned profile is within 5%(2%+3%) of the measured profile, regardless of the gradient.

## CONCLUSION

In this study we tested the use of EPID in the regular frame averaging configuration, and demonstrated satisfactory results for verification of IMRT delivery. Although the upgrade being developed by the vender will account for the charges unaccounted by the current configuration, our results indicate that, for clinical IMRT fields, the errors are either insignificant, or can be easily corrected without the upgrade. Thus, the use of the current frame averaging configuration is quite acceptable in the clinical environment; the vender's upgrade, on the other hand, is not absolutely necessary and may be cost ineffective.
